# Examining the Association Between Exposure to the #ShesWell Campaign and Black Women’s Conversations with Healthcare Providers About Pre-Exposure Prophylaxis (PrEP)

**DOI:** 10.3390/ijerph22081224

**Published:** 2025-08-06

**Authors:** Vanessa Boudewyns, Gabriel Madson, Stefanie K. E. Anderson, Hannah Getachew-Smith, Ryan S. Paquin, Sarah E. Sheff, Nivedita L. Bhushan, Revae S. Downey, Jennifer D. Uhrig

**Affiliations:** 1 RTI International, Research Triangle Park, NC 27709, USArpaquin@rti.org (R.S.P.); ssheff@rti.org (S.E.S.); nbhushan@rti.org (N.L.B.); uhrig@rti.org (J.D.U.); 2 Centers for Disease Control and Prevention, Atlanta, GA 30333, USA

**Keywords:** HIV prevention, women and PrEP, pre-exposure prophylaxis, black women, campaign evaluation, public health messages, healthcare providers

## Abstract

Low uptake of pre-exposure prophylaxis (PrEP) for HIV prevention among Black women has been partly attributed to barriers related to patient-provider communication. The goal of this paper was to investigate the association between exposure to the #ShesWell campaign and Black women’s communication about PrEP with a healthcare provider (HCP). We conducted a cross-sectional survey of 403 sexually active, Black women after the initial phase of #ShesWell and used multivariable regression models to analyze whether exposure to #ShesWell was associated with talking to an HCP about PrEP or intention to discuss PrEP with an HCP in the future. Approximately 33% of women surveyed reported exposure to #ShesWell. Campaign exposure was significantly associated with talking to an HCP in the past year about PrEP (OR = 4.96, *p* = 0.001) and intention to discuss PrEP with an HCP in the next six months (*B* = 0.29, *p* = 0.038). Stronger beliefs that doctors should initiate sexual health conversations were positively associated with past PrEP conversations (OR = 2.32, *p* < 0.001) and future intention (*B* = 0.11, *p* = 0.029). Greater comfort discussing prevention (*B* = 0.35, *p* < 0.001), self-efficacy discussing PrEP (*B* = 0.29, *p* = 0.001), and concern about getting HIV (*B* = 0.51, *p* < 0.001) were also associated with intention to discuss PrEP with an HCP. Findings highlight the potential for communication campaigns to motivate patient-provider communication about PrEP, addressing a reported barrier to PrEP uptake among Black women.

## 1. Introduction

Although Black/African American women (hereafter, Black women) are disproportionately impacted by HIV in the United States, women represent fewer than 5% of pre-exposure prophylaxis (PrEP) users nationally, and Black women comprise fewer than 1% of users for whom race/ethnicity data are available [1,2,3]. Low PrEP uptake among Black women can be attributed, in part, to barriers at both the patient and healthcare provider (HCP) levels that limit conversations about PrEP and inhibit prescriptions [4,5,6,7,8].

Barriers to PrEP uptake among women include low awareness of PrEP, anticipated stigma or negative stereotypes about PrEP users (e.g., that they are promiscuous or HIV-positive), and discomfort initiating conversations with HCPs about sexual health, all of which have been associated with reluctance to discuss PrEP with HCPs [9,10]. Furthermore, perceptions that family, partners, or friends would disapprove of using PrEP also negatively impact women’s willingness to engage in conversations about PrEP with HCPs [9]. Medical mistrust, stemming from historical and contemporary negative experiences with the healthcare system, can further deter Black women from discussing or considering PrEP [11,12,13,14,15,16,17]. Multiple barriers prevent HCPs from having routine conversations about PrEP with their female patients. Despite updated Centers for Disease Control (CDC) guidelines recommending that HCPs discuss PrEP with all sexually active adults, many fail to engage in such conversations, reinforcing gaps in access and equity [18]. HCPs have reported a lack of knowledge about PrEP and discomfort discussing sexual health behaviors and PrEP with patients [19,20]. Organizational constraints, such as limited appointment time and competing clinical priorities, exacerbate these barriers [19,20]. HCPs prefer that their patients initiate conversations about PrEP, yet many women prefer that HCPs begin these conversations, resulting in mutual communication challenges [21,22]. Additional qualitative research has found that HCPs in family planning centers avoid conversations about PrEP due to their discomfort addressing sensitive issues related to social determinants of health [23].

Public health communication campaigns have the potential to motivate patient-provider conversations about one’s sexual health and HIV prevention strategies, like PrEP, thereby addressing an important barrier to PrEP uptake. The CDC’s #ShesWell campaign, a component of the Let’s Stop HIV Together campaign under the Ending the HIV Epidemic in the U.S. (EHE) initiative, is a dual-audience communication campaign designed to increase demand for PrEP among Black women while strengthening HCPs’ capacity related to prescribing PrEP and counseling patients. #ShesWell was initially implemented from 15 March to 23 August 2022, in four Ending the HIV Epidemic (EHE) jurisdictions (Cuyahoga County, OH [Cleveland]; Hamilton County, OH [Cincinnati]; Duval County, FL [Jacksonville]; and Sacramento County, CA [Sacramento-Stockton-Modesto]). #ShesWell messages conveyed that PrEP is for women; used language to express a personal connection and sense of community; featured themes of empowerment, sexual autonomy, and a holistic approach to sexual health and well-being; and had clear calls to action (e.g., learn more about PrEP through the #ShesWell website, locate an HCP through the HIV.gov services locator widget, and talk to an HCP about taking PrEP). More information about #ShesWell and additional campaign materials are available online at www.cdc.gov/stophivtogether/sheiswell.

Although mass-media health campaigns can increase awareness and sometimes influence behavior, meta-analyses show their effects are often modest and variable, particularly for HIV prevention (e.g., LaCroix et al. [24] Noar et al. [25]; Snyder et al. [26]). Few studies have examined whether such campaigns can reduce interpersonal barriers and promote PrEP engagement among Black women. The present study therefore investigates whether exposure to the #ShesWell campaign was associated with Black women’s communication with HCPs about PrEP and their intentions to initiate such conversations in the future. Specifically, we focused on addressing three research questions (RQs):

RQ1: Was self-reported exposure to #ShesWell associated with having a conversation about PrEP with an HCP in the past year?
RQ2: Was self-reported exposure to #ShesWell associated with intention to have a conversation about PrEP with an HCP in the next 6 months?
RQ3: What other factors were associated with having talked to an HCP about PrEP, or reporting intention to do so in the future?

## 2. Materials and Methods

### 2.1. Sample and Data Collection

To evaluate the #ShesWell campaign, we administered a 20 min cross-sectional online survey between January and March 2023 to women residing in four EHE jurisdictions where the initial phase of #ShesWell was implemented. A non-probability–based purposive sampling strategy was used to recruit respondents via a panel aggregator, Qualtrics (Provo, UT, USA). To ensure balanced representation, we set goals of 25% of the sample from each county. Individuals were eligible for the survey if they self-identified as (1) a woman, (2) aged 18 to 64, (3) having no prior HIV diagnosis, (4) having vaginal or anal sex with at least one male partner in the past 12 months, and (5) able to complete the survey in English. We used a non-probability, purposive sampling strategy with soft quotas on age and education to ensure a diverse sample. In addition, we oversampled Black women to align with the #ShesWell campaign’s primary audience. All respondents provided online informed consent and, following standard panel practice, received the incentive advertised in their survey invitation (cash or points equivalent, capped at USD20 in accordance with OMB guidance) after completing the survey and passing data-quality checks. The project was reviewed and determined to be non-research by the Institutional Review Board at RTI International.

Respondents who consented and completed the survey but failed one or more of our quality checks were removed from the final sample. We collected 602 total completes as part of the larger study, 403 (67%) of which were Black women who were intentionally oversampled. The analyses and results presented here focus only on this subgroup of 403 Black women.

### 2.2. Measures

Outcome Variables. We used two survey items to assess communication with HCPs about PrEP (See Supplementary File S1). Note that for all items that referenced HCPs, the items intentionally used the generic term “healthcare provider” without defining specific disciplines; therefore, responses may refer to any clinician whom participants perceive as their primary point of care (e.g., physicians, nurse practitioners, physician assistants, or counselors). The first item asked participants whether they recalled having a PrEP-based conversation with their HCP in the last year (“In the past year, did you talk to a healthcare provider about PrEP?”). For analysis, we coded the measure as a binary variable, with “Yes” coded as 1 and all other outcomes (“No,” “Prefer not to answer,” “Didn’t see an HCP in the last 12 months”) coded as 0. The second survey item asked about intended PrEP conversations with one’s HCP in the future (“Please tell us how unlikely or likely it is that you’ll do the following in the next 6 months: Talk to a healthcare provider about PrEP”). Responses ranged from 1 = very unlikely to 5 = Very likely.

Predictor Variables. Our primary predictor variable was self-reported campaign exposure based on aided recall of the #ShesWell campaign and measured using a binary variable. During the survey, respondents viewed a series of image collages featuring campaign ads and were asked whether they had seen the corresponding campaign. Two of the collages (depicting the two style variations in ads #ShesWell used) displayed images from #ShesWell. To reduce bias and mask the purpose of the evaluation, the survey also included filler collages made up of images from other past and present PrEP campaigns that participants were unlikely to have seen. Respondents who recognized the #ShesWell ads were coded as exposed (1); all others were coded as not exposed (0).

Guided a priori by behavioral theory and expert consultation, we also measured several other constructs thought to have a theoretical influence on communicating with an HCP about PrEP [7,10,14,27,28,29]. Each of these variables were included as predictors in our multivariable models. The predictors included five constructs measured on five-point agreement scales (1 = Strongly disagree to 5 = Strongly agree): belief about the role of HCPs in initiating conversations about sexual health (“I believe that the doctor is mostly responsible for starting the conversation about sexual health with the patient”), comfort talking to HCPs about HIV prevention options (“I am comfortable talking to my healthcare provider about my HIV prevention options”), perceptions of whether their HCP respects them (“My healthcare provider respects me”), self-efficacy toward initiating conversations with an HCP about PrEP (“If I wanted to, I could talk to my healthcare provider about PrEP”), and importance of sexual health (“My sexual health matters to me”). Additionally, we included an item that measured concern about getting HIV (“Keeping in mind the different ways people can get HIV, how concerned are you about you, personally, getting HIV”), with a four-point response scale ranging from 1 = Not at all concerned to 4 = Very concerned.

**Control Variables.** Socio-demographic variables in the models were selected a priori and included self-reported measures of age, educational status, income, and whether respondents had health insurance.

### 2.3. Statistical Analyses

All data cleaning and statistical analyses were performed in R Core Team (2023) Version 4.4.1. We began by calculating descriptive statistics for all outcomes and predictor variables (e.g., frequencies, means, correlations). This preliminary analysis allowed us to assess potential pitfalls in our statistical models (e.g., multicollinearity, normality violations). Next, we ran multivariable models to address the research questions. To address RQ1, we estimated a binary logistic regression model to assess whether exposure to the #ShesWell campaign, along with other beliefs and sociodemographic predictor variables, were associated with having a conversation about PrEP with an HCP in the past year. To address RQ2, we estimated an ordinary least squares (OLS) regression to assess whether exposure to the campaign, along with other predictor variables, were associated with intention to have a conversation about PrEP with an HCP in the next 6 months. Due to the non-normal distribution of the outcome variable, we also ran an ordinal logistic regression model where intended discussions about PrEP were treated as ordinal, rather than pseudo-continuous. Results from that model suggested similar conclusions to the OLS regression results. To address RQ3, we assessed whether each predictor variable was significantly associated with either past conversations or intention for future conversations with an HCP about PrEP, controlling for exposure, in both models. We assessed multicollinearity in two ways [30]. First, using listwise deletion procedures, we examined Pearson correlation coefficients of all predictor and outcome variables prior to running the multivariable models. We considered variables with a 0.70 or higher correlation a threat of multicollinearity. Next, we examined the variance inflation factor (VIF) of each coefficient in all statistical models and considered any variable with a parameter estimate value of ten or higher a threat of multicollinearity. Based on an examination of the correlation coefficients and the VIF of each coefficient, we found no strong evidence of multicollinearity [30].

## 3. Results

### 3.1. Respondent Characteristics, Campaign Exposure and Communication with HCPs About PrEP

Characteristics of the sample are provided in Table 1. More than two thirds of respondents (68.3%) were 18–44 years of age (M = 37.92; SD = 11.45; range: 18–64), and roughly one third had a high school education or less and an annual income of less than USD20,000; most respondents (94.5%) reported having health insurance. One third of respondents (33.0%) reported exposure to #ShesWell. Only 10.7% of respondents talked to an HCP about PrEP in the past year and were somewhat likely to intend to talk to an HCP about PrEP in the next 6 months (M = 3.40; SD = 1.41).

Discussing PrEP with an HCP in the past year was significantly associated with stronger agreement that their HCP respected them (*r* = 0.11), stronger agreement that HCPs are responsible for initiating sexual health conversations (*r* = 0.25), and exposure to #ShesWell (*r* = 0.25) (all *p* < 0.05; see Table 2). Additionally, intention to discuss PrEP with an HCP in the next six months was significantly correlated with stronger agreement that their HCP respected them (*r* = 0.17), stronger agreement that doctors are responsible for initiating sexual health conversations (*r* = 0.14), comfort in talking with an HCP about HIV prevention options (*r* = 0.24), self-efficacy initiating a conversation about PrEP with an HCP (*r* = 0.21), and exposure to #ShesWell (*r* = 0.14) (all *p* < 0.01).

### 3.2. Communicating with HCP About PrEP

After controlling for relevant demographics and other variables thought to affect either exposure to #ShesWell or PrEP discussion with an HCP, exposure to the #ShesWell campaign was positively and significantly associated with having discussed PrEP with an HCP in the past year (OR = 4.96, *p* = 0.001), providing supporting evidence for RQ1 (see Table 3). Looking at the average marginal effect of campaign exposure, we found a 9.93 percentage point increase in the probability of having a conversation with an HCP about PrEP in the past year for those exposed to #ShesWell: Among women exposed to #ShesWell, 28.3% reported having had a conversation with an HCP about PrEP versus 18.4% among those who were not exposed to the campaign. We also found that stronger agreement with the belief that doctors are responsible for initiating conversations about sexual health was positively associated with having discussed PrEP with an HCP in the past year (OR = 2.32, *p* < 0.001); none of the other covariates in the model were statistically significant.

### 3.3. Intention to Discuss PrEP with HCP

Providing support for RQ2, we found that exposure to #ShesWell was positively and significantly associated with the intention to discuss PrEP with an HCP in the next 6 months (*B* = 0.29, *p* = 0.038), with a marginal effect of about one third of a point on the five-point likelihood scale (see Table 4).

We also found that stronger agreement with the belief that doctors are responsible for initiating conversations about sexual health (*B* = 0.11, *p* = 0.029), comfort discussing HIV prevention options with an HCP (*B* = 0.35, *p* < 0.001), greater self-efficacy in discussing PrEP with an HCP (*B* = 0.29, *p* = 0.001), and greater concern about getting HIV (*B* = 0.51, *p* < 0.001) were significantly and positively associated with intention to discuss PrEP with an HCP in the next 6 months. Conversely, stronger agreement that one’s sexual health mattered to them was negatively associated with intention for future discussions of PrEP with an HCP (*B* = −0.30, *p* = 0.001); none of the other predictors in the model were statistically significant.

We explored this finding further and found the hallmarks of classical suppression were evident in this case [31], suggesting that agreement that one’s sexual health matters was a potential suppressor of the relationships between both comfort and self-efficacy on intention to discuss PrEP with an HCP. Specifically, the bivariate correlations for (1) my sexual health matters and intention are insignificant (*r* = −0.06, *p* = 0.243), (2) comfort talking about HIV prevention options and self-efficacy to initiate conversations on intention are positive (*r* = 0.24, *p* < 0.001 and *r* = 0.21, *p* < 0.001, respectively), and (3) comfort and self-efficacy on my sexual health matters are positive (*r* = 0.27, *p* < 0.001 and *r* = 0.19, *p* < 0.001, respectively). Further, if the belief “my sexual health matters” is removed from the model, the regression coefficients for comfort talking about HIV prevention options (*B* = 0.29, *SE* = 0.09, β = 0.17) and self-efficacy to initiate conversations (*B* = 0.26, *SE* = 0.09, β = 0.15; full model not reported) become smaller. The natural indirect effect (NIE) derived from these models for comfort talking about HIV prevention options on intention to discuss PrEP with an HCP was significantly less than 0 (NIE = −0.06, *SE* = 0.02, *p* = 0.016, 95% CI_bias-corrected_ [−0.15, −0.01]), while its controlled direct effect (CDE) was positive (*B* = 0.35, *SE* = 0.09, *p* < 0.001, 95% CI_bias-corrected_ [0.15, 0.57]), indicating that “my sexual health matters to me” is a suppressor of this relationship [32]. However, the NIE of self-efficacy to initiate conversations on intention to discuss PrEP with an HCP was not significantly less than 0 (NIE = −0.03, *SE* = 0.02, *p* = 0.089, 95% CI _bias-corrected_ [−0.09, 0.00]). As such, adding “my sexual health matters” to the model accounts for unexplained variance in the association between comfort and intention to discuss PrEP with an HCP but not between self-efficacy and intention.

## 4. Discussion

This study aimed to investigate the association between exposure to the #ShesWell campaign and Black women’s communication with HCPs about PrEP for HIV prevention. Exposure to the campaign was significantly associated with past discussions and future intention to discuss PrEP with HCPs, suggesting a potential association between campaign exposure and PrEP-related conversations, which warrants further causal investigation. Interpersonal discussion triggered by campaigns is an established mechanism linking media exposure to health outcomes, particularly for intimate or stigmatized issues such as HIV prevention. Our findings align with this mechanism, showing that women exposed to #ShesWell were substantially more likely to talk with their providers about PrEP [33].

Our findings also highlight the association between believing that HCPs should initiate sexual health conversations and reported communication about PrEP. Women who strongly endorsed this belief were more likely to report both past and intended PrEP communication with HCPs. In line with prior studies [10,34], it is possible that women who think it is their HCP’s responsibility to bring up sexual health topics might be more comfortable in the clinical setting and trust their HCPs more, thereby facilitating an open dialog about PrEP. This finding may also reflect not only women’s preferences but also their prior experiences. Specifically, Black women whose HCPs initiated respectful, informative conversations about PrEP may have used that experience to form beliefs about such discussions. As Irie and Blackstock [18] suggest, HCPs can act as gardeners, planting seeds through early, respectful PrEP conversations, even when uptake is not immediate, creating a foundation for future engagement. Therefore, further exploration into the best practices for supporting HCPs in initiating such conversations could be beneficial [35].

Comfort discussing HIV prevention options and self-efficacy in initiating PrEP-related conversations were also both positively correlated with intention to discuss PrEP, highlighting the importance of these factors in HIV communication campaigns. It may be that higher levels of comfort and self-efficacy are necessary for women to be receptive to HCP-initiated conversations about PrEP [28,36]. Communication campaigns might consider including message components that not only encourage talking to a doctor about PrEP, but also provide resources to boost women’s confidence and self-efficacy in engaging in such discussions. One example of this is the “Risk to Reasons” initiative from ViiV Healthcare, which includes an activity book that features interactive exercises, games, and conversation prompts to help Black women explore their desires, prepare for HCP interactions, and practice asking for PrEP for the first time “https://viivhealthcare.com/en-us/supporting-the-hiv-community/positive-action/risk-to-reasons/” (accessed on 10 June 2025).

We also found that women who were more concerned about personally getting HIV were more inclined to engage in conversations about PrEP. This aligns with a body of previous research suggesting that perception of risk can be a significant motivator in PrEP-related behaviors and discussions [19,36,37,38]. It is important to note, however, that research consistently indicates a tendency among individuals to underestimate their risk of acquiring HIV [39,40]. This can create a barrier to engaging in PrEP-related discussions, as those who perceive their risk as low may not see the value in discussing or using PrEP [17]. Messaging that emphasizes that PrEP is an option for anyone interested in additional preventive measures, not just for those with a high perceived risk of HIV, along with empowering Black women to engage in protective behavior, may be associated with greater PrEP uptake [36].

Interestingly, affirming the importance of one’s sexual health acted as a suppressor variable, potentially masking or modifying the bivariate relationship between comfort in discussing HIV prevention options and intention to discuss PrEP with an HCP. One explanation is that women who perceive their sexual health to be important could be more confident in their current preventive measures and thereby less inclined to discuss PrEP. Future research should probe this association further to better understand the nuanced relationship between perceived importance of sexual health and intention to discuss PrEP with an HCP.

Combined with results from the broader evaluation of #ShesWell that found that exposure to the campaign was associated with greater PrEP awareness and knowledge (unpublished data), as well as attitudinal beliefs, norms, perceived behavioral control, and intention to take PrEP [41], these findings have public health relevance. Not only do these findings provide data-based insights into motivating factors that might help Black women feel supported and empowered to discuss and access PrEP, but they suggest that campaigns like #ShesWell may be associated with greater awareness and dialog about PrEP use and intention to use PrEP. For HCPs, these results echo calls regarding the importance of being proactive about initiating conversations about sexual health, especially with populations disproportionately impacted by HIV [18].

There were a number of strengths to this study, including the campaign design that incorporated effective principles such as formative research, message targeting, use of selective channels with optimal reach, and evaluation of exposure and outcomes [25]. Nonetheless, this study bears a few potential limitations that should be considered when interpreting the findings. First, the cross-sectional design prohibits us from establishing causality between campaign exposure and subsequent discussions about PrEP. For example, while our findings show that believing HCPs should initiate PrEP conversations was associated with PrEP conversations, we cannot determine whether that belief led to the conversation or whether having a positive experience with an HCP shaped the belief. In terms of shaping future campaigns, these results suggest that a dual approach—reaching both patients and HCPs—may be effective in facilitating communication. Second, because we relied on a non-probability, quota-based online panel, the sample may not fully represent all Black women in the four EHE counties or nationally. Third, aided recall of campaign exposure may suffer from misattribution, given the existence of other PrEP campaigns. Additionally, the study relied on self-reported data, which might be influenced by social desirability bias, especially for sensitive topics such as sexual health and HIV prevention. Finally, because the survey did not differentiate between provider types, we were unable to examine whether campaign effects vary by provider cadre. Future studies should explore whether conversations about PrEP differ when initiated with physicians versus other health professionals.

## 5. Conclusions

The current article addresses a gap in the literature on the effectiveness of communication campaigns on increasing intention to talk to HCPs about PrEP, particularly among Black women. Our study highlights a potential association between exposure to public health campaigns like #ShesWell and PrEP-related discussions among Black women. More research should examine the role of health communication in reducing barriers to patient-provider communication to support the uptake of PrEP. Further efforts to scale up such campaigns and encourage HCPs to proactively discuss PrEP with patients may help reduce the disproportionately high rates of HIV among Black women.

## Figures and Tables

**Table 1:** #ShesWell campaign survey respondent characteristics, campaign exposure, and communication with HCPs about PrEP, January to March 2023 (*N* = 403).

Characteristics	*N*	%
Age		
18–24 years	60	14.9
25–44 years	215	53.4
45–64 years	128	31.8
Education		
High school education or less	135	33.5
Some college	164	40.7
College graduate	104	25.8
Income		
USD20,000 or less	135	33.7
USD20,001 to USD40,000	135	33.7
USD40,001 to USD75,000	80	20.0
More than USD75,000	51	12.7
Health Insurance		
Yes	381	94.5
No	22	5.5
Heard/seen any #ShesWell Campaign ads	133	33.0
Talked to HCP about PrEP in past year (Yes)	43	10.7
Intention to talk to HCP about PrEP in next 6 months (Mean, SD)	3.40	1.41

Note: Intention to talk to HCP ranged from 1 = Very unlikely to 5 = Very likely.

**Table 2:** Bivariate correlations of predictor and outcome variables among black women in the United States, January–March 2023 (*N* = 368).

Variable	1	2	3	4	5	6	7	8	9	10	11	12	13
1. Talked to HCP about PrEP in past year	–												
2. Intention to talk to HCP about PrEP in next 6 months	0.09	–											
3. Exposed to #ShesWell	0.25 ***	0.14 **	–										
4. My HCP respects me	0.11 *	0.17 ***	0.07	–									
5. Doctor is mostly responsible for starting the conversation about sexual health with the patient	0.25 ***	0.14 **	0.08	0.13 *	–								
6. I am comfortable talking to my HCP about my HIV prevention options	0.03	0.24 ***	−0.01	0.46 ***	0.07	–							
7. If I wanted to, I could talk to my healthcare provider about PrEP	0.08	0.21 ***	0.10	0.31 ***	−0.13 *	0.33 ***	–						
8. My sexual health matters to me	0.02	−0.06	0.01	0.16 **	−0.02	0.27 ***	0.19 ***	–					
9. Concern about personally getting HIV	−0.04	0.43 ***	0.05	0.03	0.06	0.02	0.01	0.00	–				
10. Age	−0.10	−0.03	−0.15 **	0.09	−0.12 *	0.04	0.10	0.01	0.02	–			
11. Education ^a^	−0.15 **	−0.10 *	−0.03	0.01	−0.08	0.01	0.05	−0.01	−0.10	0.21 ***	–		
12. Income ^a^	−0.12 *	−0.09	0.01	−0.03	0.07	−0.10	0.01	−0.11 *	−0.07	0.19 ***	0.49 ***	–	
13. Health insurance	−0.02	0.06	0.02	0.05	0.02	−0.01	0.00	0.00	0.05	0.06	0.02	0.08	–
Mean	0.08	3.40	0.31	4.35	2.72	4.44	4.23	4.68	2.45	37.92	–	–	0.94
SD	0.27	1.41	0.46	0.78	1.30	0.81	0.82	0.71	1.08	11.45	–	–	0.23

– = not applicable. Spearman’s rank-order correlation, ^a^, is reported for associations with education and income. All other values are Pearson’s correlation coefficients, *r*. *** *p* < 0.001; ** *p* < 0.01; * *p* < 0.05.

**Table 3:** Binary logistic regression results on black women talking to an HCP in the past year about PrEP in the U.S., January–March 2023 (*N* = 367).

	OR	SE	*z*	*p*	95% CI
Exposed to #ShesWell	4.96	2.36	3.36	0.001	(1.95, 12.62)
My HCP respects me	1.54	0.70	0.95	0.340	(0.63, 3.77)
Doctor is mostly responsible for starting the conversation about sexual health with the patient	2.32	0.47	4.17	<0.001	(1.56, 3.45)
I am comfortable talking to my HCP about my HIV prevention options	0.63	0.24	−1.21	0.227	(0.30, 1.33)
If I wanted to, I could talk to my healthcare provider about PrEP (self-efficacy)	1.59	0.55	1.33	0.182	(0.80, 3.15)
My sexual health matters to me	0.99	0.37	−0.03	0.976	(0.47, 2.07)
Concern about personally getting HIV	0.74	0.16	−1.35	0.177	(0.48, 1.14)
Age	0.98	0.02	−0.88	0.378	(0.94, 1.02)
Education (ref = High school education or less)					
Some college	0.99	0.55	−0.02	0.984	(0.33, 2.97)
College graduate	0.38	0.31	−1.18	0.239	(0.07, 1.92)
Income (ref = USD20,000 or less)					
USD20,001 to USD40,000	0.51	0.30	−1.16	0.247	(0.16, 1.60)
USD40,001 to USD75,000	0.70	0.48	−0.51	0.609	(0.18, 2.70)
More than USD75,000	0.18	0.22	−1.37	0.169	(0.02, 2.08)
Health insurance (ref = No)					
Yes	0.79	0.68	−0.28	0.781	(0.14, 4.29)
Intercept	0.00	0.01	−2.26	0.024	(0.00, 0.47)

CI = confidence interval; HCP = healthcare provider; OR = odds ratio; PrEP = pre-exposure prophylaxis; SE = standard error. Note: age, education, income, and health insurance variables are included as demographic controls that may influence propensity to exposure of the campaign as well as general discussions with an HCP. All other model variables are included due to their potential confounding nature with either the explanatory (campaign exposure), outcome variable, or both. Model Statistics: Predicted Class Probabilities = 0.94; Proportional Reduction in Error = 0.28; Max VIF = 1.81; unadjusted results of campaign exposure: OR = 5.76, *p* < 0.001.

**Table 4:** Ordinary least squares regression results on Black women’s intention to discuss PrEP with an HCP in the next six months, January-March 2023 (*N* = 366).

	*B*	*SE*	95% CI	β	*t*	*p*
Campaign awareness	0.29	0.14	(0.02, 0.57)	0.10	2.08	0.038
My HCP respects me	0.04	0.09	(−0.15, 0.22)	0.02	0.37	0.710
Doctor is mostly responsible for starting the conversation about sexual health with the patient	0.11	0.05	(0.01, 0.21)	0.10	2.20	0.029
I am comfortable talking to my HCP about HIV prevention options	0.35	0.09	(0.17, 0.53)	0.20	3.77	<0.001
If I wanted to, I could talk to my healthcare provider about PrEP (self-efficacy)	0.29	0.09	(0.13, 0.46)	0.17	3.44	0.001
My sexual health matters to me	−0.30	0.09	(−0.48, −0.12)	−0.15	−3.23	0.001
Concern about personally getting HIV	0.51	0.06	(0.40, 0.63)	0.40	8.56	<0.001
Age	0.00	0.01	(−0.01, 0.01)	−0.02	−0.47	0.640
Education (ref = High school education or less)						
Some college	−0.08	0.16	(−0.39, 0.24)	−0.03	−0.48	0.629
College graduate	−0.11	0.20	(−0.50, 0.27)	−0.03	−0.58	0.563
Income (ref = USD20,000 or less)						
USD20,001 to USD40,000	−0.03	0.16	(−0.35, 0.29)	−0.01	−0.19	0.846
USD40,001 to USD75,000	−0.18	0.20	(−0.57, 0.21)	−0.05	−0.93	0.353
More than USD75,000	−0.20	0.23	(−0.65, 0.25)	−0.05	−0.86	0.389
Health insurance (ref = No)						
Yes	0.28	0.27	(−0.26, 0.81)	0.05	1.02	0.308
Intercept	0.19	0.65	(−1.08, 1.46)	na	0.30	0.766

CI = confidence interval; HCP = healthcare provider; na = not applicable; PrEP = pre-exposure prophylaxis; SE = standard error. Note: *R*^2^ = 0.31; Max VIF = 1.81; unadjusted results of campaign exposure: *B* = 0.40, *p* = 0.008; see Supplementary Table S1 for results from a robust Ordinal Logistic Regression model that accounts for OLS assumptions for a five-point continuous outcome variable.

## Data Availability

Due to the nature of this research, participants of this study did not agree for their data to be shared publicly, so supporting data are not available.

## Supplementary Materials

The following supporting information can be downloaded at https://www.mdpi.com/article/10.3390/ijerph22081224/s1, Supplementary File S1: Screener and Survey Items.

## Abbreviations

The following abbreviations are used in this manuscript:

CDC	Centers for Disease Control and Prevention
EHE	Ending the HIV Epidemic
HCP	Healthcare providers
HIV	Human Immunodeficiency Virus
PrEP	Pre-exposure prophylaxis
SD	Standard Deviation
SE	Standard Error
VIF	Variance inflation factor
