# Supplementary material for: Examining the Association Between Exposure to the #ShesWell Campaign and Black Women’s Conversations with Healthcare Providers About Pre-Exposure Prophylaxis (PrEP)
Source: Int J Environ Res Public Health. 2025 Aug 6;22(8):1224. doi: 10.3390/ijerph22081224 (PMC12385958; doi:10.3390/ijerph22081224)
Supplement: Supplementary file 1 [file ijerph-22-01224-s001.zip › ijerph-3727178-supplementary.pdf]

Supplementary File A: Screener and Survey Items

NOTE: ANY TEXT IN RED WAS NOT SHOWN TO PARTICIPANTS

[SCREENER]

[SCRIPT] We're asking people to take a survey. This survey will help us learn more about what people think about HIV prevention.

RTI International, a research company, is conducting the survey. The Centers for Disease Control and Prevention (CDC) is supporting this research.

If you qualify and choose to take part in the survey, you will be compensated the amount you agreed upon before you entered into the survey

To see if you are a good match for taking this survey, we'll need to ask you some personal questions. Some of the questions will be about your sexual orientation, recent sexual activity, and your HIV status, if you know it.

It's your choice to answer the questions. You can refuse to answer any question or stop at any time. Your answers will be kept as private as possible, as allowed by law.

May we ask you the questions to see if you're a good match for taking this survey?

- ☐ Yes [CONTINUE]
- ☐ No [IMMEDIATELY TERMINATE]

Background Information

S1. How old are you?

Age__________ [INELIGIBLE IF UNDER 18 OR OVER 64]

- ☐ Don't know [INELIGIBLE]
- ☐ Prefer not to answer [INELIGIBLE]

S2. In what ZIP Code do you currently live? *This information is being collected to answer questions about where people live. The information you share will be kept strictly private.*

______________ (5 digits only)

- ☐ Prefer not to answer [INELIGIBLE]

S3. What sex were you assigned at birth on your original birth certificate?

- ☐ Male [INELIGIBLE]
- ☐ Female
- ☐ Don't know [INELIGIBLE]
- ☐ Prefer not to answer [INELIGIBLE]

S4. Do you currently describe yourself as male, female, or transgender?

- ☐ Male [INELIGIBLE]
- ☐ Female
- ☐ Transgender [INELIGIBLE]
- ☐ None of these [INELIGIBLE]
- ☐ Prefer not to answer [INELIGIBLE]

S5. What is your race or ethnic background?

[PROGRAMMING NOTE: PARTICIPANTS WILL BE ABLE TO SKIP THESE QUESTIONS, BUT IF THEY DO, THEY WILL FIRST SEE A PROMPT ASKING THEM TO FILL OUT THE QUESTION AND IF THEY STILL CHOOSE TO SKIP IT, THEY WILL BE TERMINATED]

S5.1. *Race* SELECT ONE OR MORE

- ☐ African American or Black
- ☐ American Indian or Alaska Native
- ☐ Asian
- ☐ Native Hawaiian or Other Pacific Islander
- ☐ White

S5.2. *Ethnicity* SELECT ONE

- ☐ Hispanic or Latino
- ☐ Not Hispanic or Latino

S6. Which of the following best describes how you think of yourself?

- ☐ Gay (lesbian or gay)
- ☐ Straight, that is, not gay or lesbian
- ☐ Bisexual
- ☐ Something else
- ☐ I don't know the answer
- ☐ Prefer not to answer

S7. Did you have vaginal or anal sex with a **male** partner in the past 12 months?

- ☐ Yes
- ☐ No [INELIGIBLE]
- ☐ Don't know [INELIGIBLE]
- ☐ Prefer not to answer [INELIGIBLE]

S8. Have you ever been tested for HIV? An HIV test checks whether someone has the virus that causes AIDS.

- ☐ Yes [Go to S9]
- ☐ No [Go to S10]
- ☐ Prefer not to answer [INELIGIBLE]

S9. What was the result of your most recent HIV test?

- ☐ I tested positive for HIV [INELIGIBLE]
- ☐ I tested negative for HIV
- ☐ My results were unclear
- ☐ I never got my results/Don't know
- ☐ Prefer not to answer [INELIGIBLE]

S10. How often did you and your **male** sexual partner(s) use condoms for vaginal and/or anal sex in the past 12 months?

- ☐ Never [Go to S12]
- ☐ Occasionally [Go to S12]
- ☐ Usually [Go to S12]
- ☐ Always [Go to S11]
- ☐ Don't know [Go to S12]
- ☐ Prefer not to answer [INELIGIBLE]

S11. In the past 12 months, did you ever, even one time, have sex without a condom with a **male** sexual partner(s)?

- ☐ Yes
- ☐ No
- ☐ Don't know
- ☐ Prefer not to answer

S12. In the past 12 months, how many ***male*** **casual sexual partners** did you have? By casual sexual partner, we mean somebody who you **do not** think of as your spouse, boyfriend, significant other, or life partner.

- ☐ 0
- ☐ 1
- ☐ 2 to 5
- ☐ 6 to 10
- ☐ 11 to 15
- ☐ 16 to 20
- ☐ More than 20
- ☐ Prefer not to answer [INELIGIBLE]

S13. Have you had a main sexual partner within the past 12 months? By main partner, we mean somebody you would call your spouse, boyfriend, significant other, or life partner.

- ☐ Yes
- ☐ No [Go to S16]
- ☐ Prefer not to answer [INELIGIBLE]

S14. Is your main partner male, female, or transgender?

- ☐ Male
- ☐ Female
- ☐ Transgender
- ☐ None of these
- ☐ Prefer not to answer

S15. What is your main partner's HIV status?

- ☐ My main partner is HIV negative
- ☐ My main partner is HIV positive
- ☐ My main partner has not been tested for HIV
- ☐ Don't know, my main partner has not told me their HIV status
- ☐ Prefer not to answer

S16. What is the highest level of education you've completed?

- ☐ Grade school
- ☐ Less than high school graduate/some high school
- ☐ High school graduate or completed GED
- ☐ Some college or technical school
- ☐ Received four-year college degree
- ☐ Some post-graduate studies
- ☐ Received advanced degree
- ☐ Other [Specify:_______________________________]
- ☐ Prefer not to answer

If eligible – Invitation:

SCRIPT: Thank you for answering these questions. You qualify to take part in the survey.

Would you like to take part in this survey?

- ☐ Yes [CONTINUE]
- ☐ No [INELIGIBLE]

[MAIN SURVEY ITEMS]

[PrEP CONVERSATIONS WITH HCP]

In the past year, did you see a healthcare provider for any reason?

- ☐ Yes [SKIP LOGIC: GO TO NEXT QUESTION]
- ☐ No
- ☐ Prefer not to answer

In the past year, did you talk to a healthcare provider about PrEP?

- ☐ Yes
- ☐ No
- ☐ Prefer not to answer

[INTENTIONS TO TALK]

SCRIPT: Please tell us how unlikely or likely it is that you'll do the following in the next 6 months.

Talk to my healthcare provider about PrEP.

- ☐ Very unlikely
- ☐ Somewhat unlikely
- ☐ Neither unlikely nor likely
- ☐ Somewhat likely
- ☐ Very likely
- ☐ Prefer not to answer

[BELIEFS ABOUT SEXUAL HEALTH]

SCRIPT: Please tell us how much you disagree or agree with the following statement about your sexual health.

There are no right or wrong answers. Please be as honest as you can when answering the questions.

My sexual health matters to me.

- ☐ Strongly disagree
- ☐ Disagree
- ☐ Neither disagree nor agree
- ☐ Agree
- ☐ Strongly agree
- ☐ Prefer not to answer

Please tell us how much you disagree or agree with the following statements.

	**Strongly disagree**	**Disagree**	**Neither disagree nor agree**	**Agree**	**Strongly agree**	**Prefer not to answer**
My healthcare provider respects me	☐	☐	☐	☐	☐	☐
I'm comfortable talking to my healthcare provider about HIV prevention options	☐	☐	☐	☐	☐	☐
I believe that the doctor is mostly responsible for starting the conversation about sexual health with the patient	☐	☐	☐	☐	☐	☐

[CAMPAIGN RECOGNITION]

SCRIPT: We'd now like to ask you some questions about advertising campaigns that you may or may not have seen over the past year. We've grouped together some example pictures from multiple ads from different campaigns. **Please review each group of pictures and tell us if you**

remember seeing any ads from that campaign. We don’t expect you to have seen all the campaigns.

SCRIPT: Below is a collection of pictures from multiple ads from a campaign.
[PROGRAMMING NOTE: A TOTAL OF 7 COLLAGES WERE SHOWN TO EACH PARTICIPANT, ONE AT A TIME. THE ORDER OF COLLAGES WAS FULLY RANDOMIZED PER PARTICIPANT. THE IDENTICAL SINGLE-ITEM QUESTION AND THREE RESPONSE OPTIONS WERE ASKED AFTER EACH COLLAGE.]

In the past year, did ever you see any of these ads (or similar ads) from this campaign?

- ☐ Yes, I definitely saw an ad from this campaign
- ☐ No, I didn’t see any ads from this campaign
- ☐ Prefer not to answer

[COLLAGE 1: SHE’S WELL GRAPHIC VERSION]

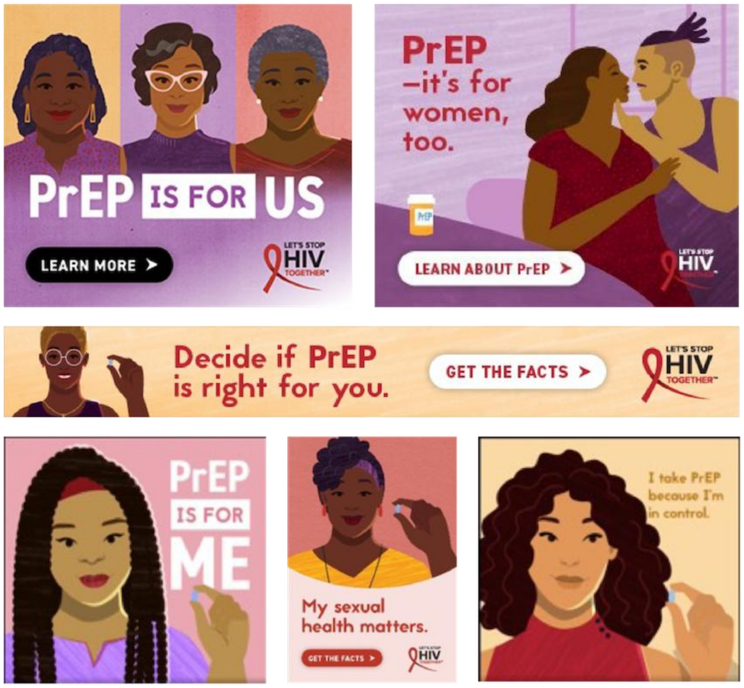

[COLLAGE 2: SHE'S WELL PHOTO VERSION]

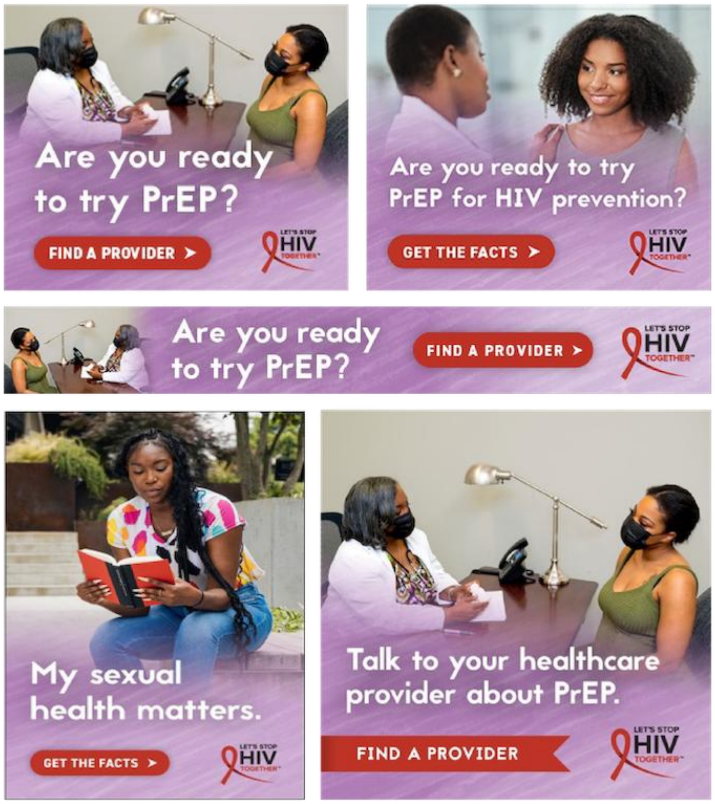

[FILLER CAMPAIGN A]

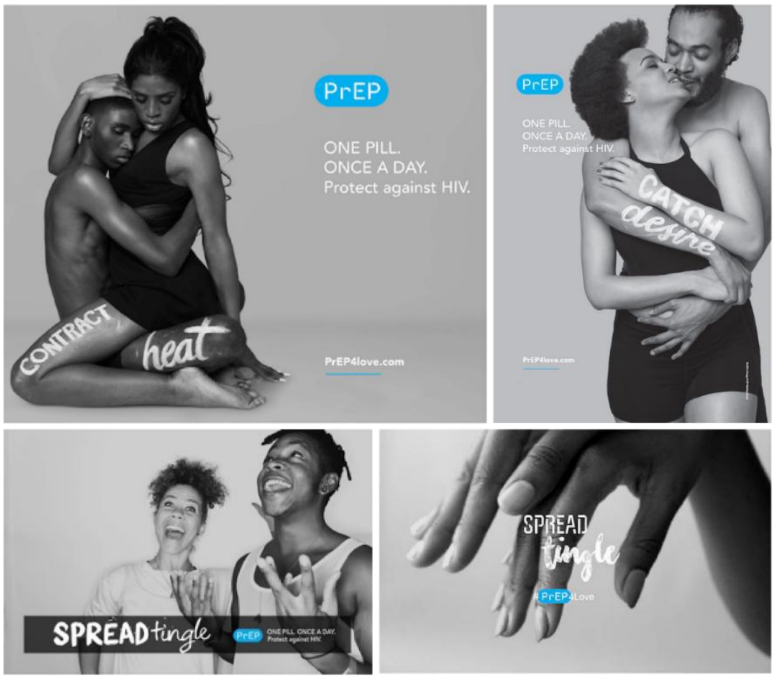

[FILLER CAMPAIGN B]

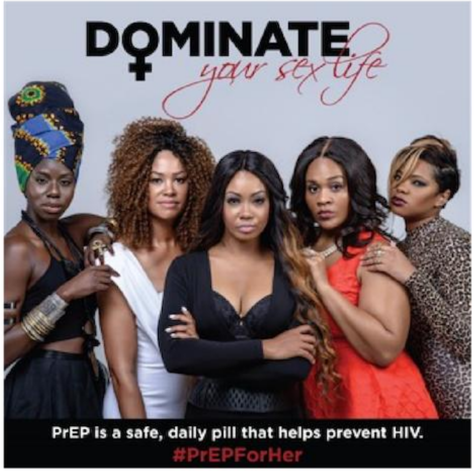

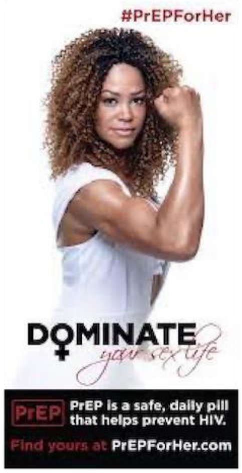

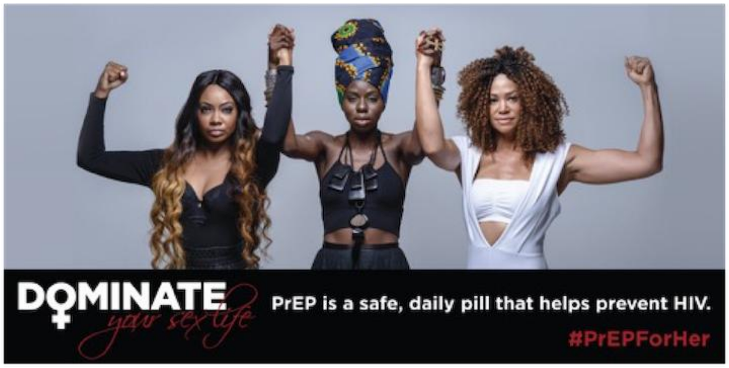

[FILLER CAMPAIGN C]

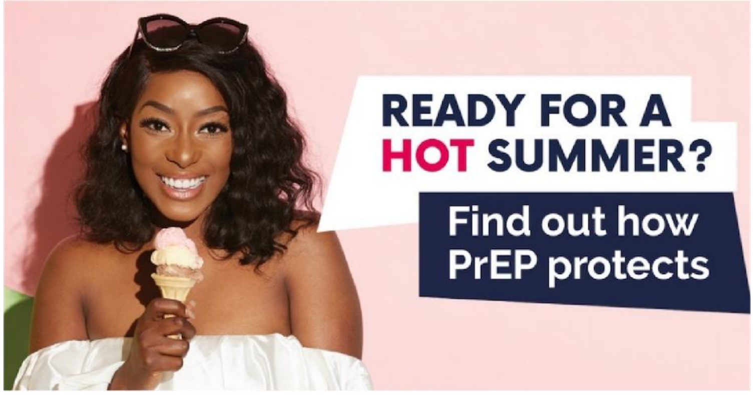

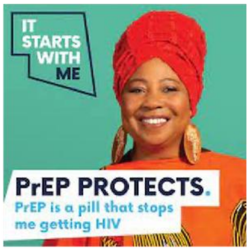

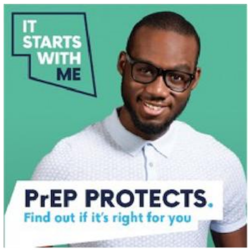

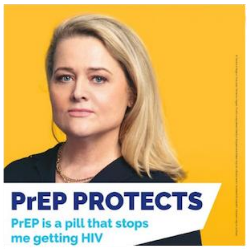

[FILLER CAMPAIGN D]

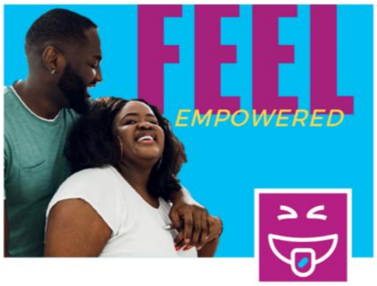

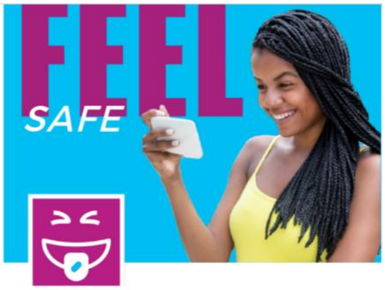

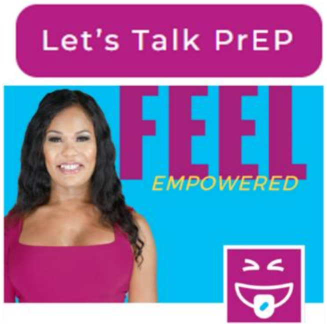

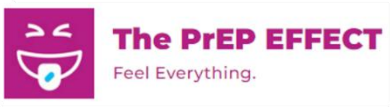

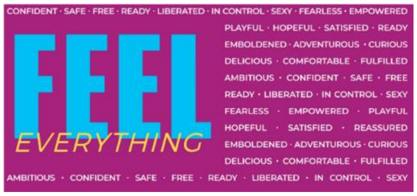

[FILLER CAMPAIGN E]

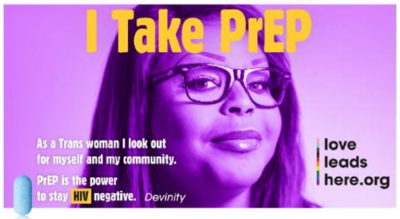

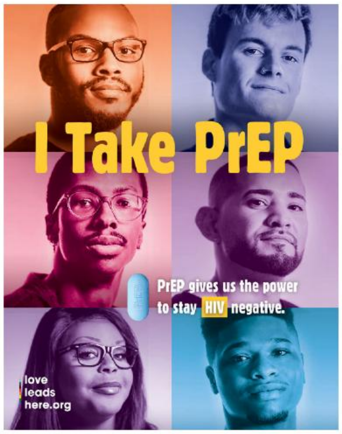

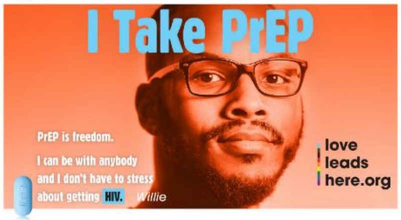

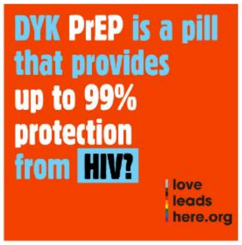

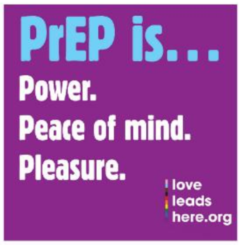

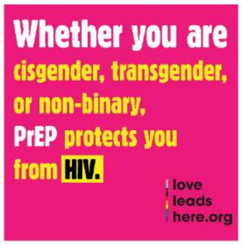

[INCOME]

Which best describes your total personal income during the past year?

- ☐ Less than $20,000
- ☐ $20,001 to $30,000
- ☐ $30,001 to $40,000
- ☐ $40,001 to $50,000
- ☐ $50,001 to $75,000
- ☐ $75,001 to $100,000
- ☐ More than $100,000
- ☐ Prefer not to answer

[HEALTH INSURANCE]

Do you currently have health insurance or healthcare coverage?

- ☐ Yes
- ☐ No
- ☐ Don't know
- ☐ Prefer not to answer

Supplementary Table S1. Ordinal Logistic Regression Results on Black Women's Intention to Discuss PrEP with an HCP in the Next Six Months, January-March 2023 (*N* = 366)

	OR	*SE*	95% CI	*t*	*p*
Campaign awareness	1.57	0.34	(1.02, 2.41)	2.06	.040
My HCP respects me	1.11	0.16	(0.84, 1.48)	0.75	.456
Doctor is mostly responsible for starting the conversation about sexual health with the patient	1.19	0.10	(1.02, 1.40)	2.14	.032
I am comfortable talking to my HCP about HIV prevention options	1.68	0.26	(1.24, 2.28)	3.38	.001
If I wanted to, I could talk to my healthcare provider about PrEP (self-efficacy)	1.65	0.22	(1.27, 2.16)	3.73	<.001
My sexual health matters to me	0.60	0.09	(0.44, 0.81)	-3.31	.001
Concern about personally getting HIV	2.20	0.22	(1.81, 2.67)	7.88	<.001
Age	0.99	0.01	(0.98, 1.01)	-0.63	.527
Education (ref = High school education or less)	0.98	0.24	(0.60, 1.59)	-0.10	.922
Some college	0.97	0.29	(0.54, 1.74)	-0.11	.912
College graduate	0.93	0.24	(0.56, 1.54)	-0.28	.780
Income (ref = $20,000 or less)	0.63	0.19	(0.35, 1.14)	-1.51	.130
$20,001 to $40,000	0.65	0.24	(0.32, 1.34)	-1.17	.243
$40,001 to $75,000	1.68	0.69	(0.75, 3.73)	1.27	.205
More than $75,000	1.57	0.34	(1.02, 2.41)	2.06	.040
Health insurance (ref = No)	1.11	0.16	(0.84, 1.48)	0.75	.456
Yes	1.19	0.10	(1.02, 1.40)	2.14	.032

CI = confidence interval; HCP = healthcare provider; na = not applicable; PrEP = pre-exposure prophylaxis; SE = standard error.

Note: Percent Correctly Predicted (PCP) =42%; Proportionate Reduction in Error (PRE) = 18%
